# Exploring the experiences of children's palliative care for forced migrant families in the United Kingdom: an interpretative phenomenological study

**DOI:** 10.3389/fped.2024.1494938

**Published:** 2025-01-06

**Authors:** Marie Clancy, Caroline Bradbury-Jones, Jenny Phillimore, Julie Taylor

**Affiliations:** ^1^ Academy of Nursing, Department of Health and Care Professions, Faculty of Health and Life Sciences, University of Exeter, Exeter, United Kingdom; ^2^ Department of Nursing and Midwifery, School of Health Sciences, College of Medicine and Health Sciences, University of Birmingham, Birmingham, United Kingdom; ^3^ School of Social Policy, Sociology and Criminology, University of Birmingham, Birmingham, United Kingdom

**Keywords:** children's palliative care, forced migrant, migration, interpretative phenomenological analysis, advisory group, refugee

## Abstract

**Introduction:**

This study focused on understanding the experiences of forced migrant families and the health care professionals who care for them within palliative care. Palliative care for children requires an active, holistic approach to care, with a focus upon improving quality of life. Forced migrant families encounter a range of additional challenges including the loss of family, belongings, and all sources of familiarity and support. The difficulties of navigating complex bureaucratic systems can confound access and communication difficulties.

**Methods:**

Interpretative Phenomenological Analysis methodology was used in this study to privilege participant perspectives and apply an active in-depth cyclical process of reflection and reflexivity. Advisory group members provided expertise in childhood illness, palliative care and forced migration, throughout the study. The Silences Framework offered novel theoretical and philosophical concepts, which helped to situate and prioritise the “silences” within the marginalised discourses of forced migration and palliative care. Seven family members and seven health care professional participants were interviewed from hospital, hospice and community palliative care settings in the UK.

**Results:**

Four overarching themes were identified related to experiences of loss and grief, communication, faith and coping strategies and alienation and discrimination. Compassionate, empathetic, family-centred care which valued family belief systems and coping strategies, optimised care. Learning with and from families was described by all participants, which enhanced understanding and fostered mutual respect. However, barriers included poor access to services and resources, protocol-led care, limited time with families, communication barriers and staff burnout.

**Discussion:**

The findings suggest the need for a specific educational pathway for palliative care professionals to include spiritual care provision, cultural humility, and moral reasoning. Interdisciplinary education including the use of lived-experience expert insights is also advocated. Sufficient time to build relationships, the importance of interpreter support and the need for better access to hospice care for forced migrant families is also recommended.

## Background

This paper presents the findings of a qualitative interpretative study into the experiences of children's palliative care for forced migrant families and those who care for them.

Currently over a 120 million people are displaced globally (1) with the effects of displacement having social, political and legal dimensions. The term forced migrant is used as an overarching term covering persons displaced across international borders and inside a single country regardless of the reasons for their displacement (2). All forced migrants share the common ground of feeling compelled to leave their homes to ensure their own and their family's survival. Sometimes these decisions are made under great duress (3). Conditions of war, political unrest and economic challenges are likely to interact and contribute to such decisions (4). The term “forced migrant” includes asylum seekers who have lodged an application for protection from persecution (5) and refugees whose asylum claim has been successful (6).

Some forced migrant families seek to come to the UK to find a diagnosis, treatment or cure for their children, with resettlement pathways sometimes open to those with children with complex medical conditions. Other times children fall sick after arriving in the UK Whenever their children become sick, families generally experience long and dangerous journeys, alongside the grief of separation from home and family. Reduced agency and feelings of disempowerment after leaving all sources of familiarity are thus common (7). Allen and Ögtem-Young (8) found that many migrants in the UK described notions of “otherness” and “foreign-ness” which led to anxiety, uncertainty and instability.

As a result of some of these combined experiences, forced migrant families often have extensive and diverse needs (9). They may have mental health conditions such as depression, isolation and grief. Trauma can result from engagement with asylum processes with problems including lack of access to essential resources such as food, accommodation, employment and health services. Mayblin et al. (10) report on what they call the slow violence of everyday life. They detail the intense harms that forced migrants experience at the hands of policy designed to ensure every aspect of daily life is difficult. They refer to the idea of necropolitics, of being “kept alive but in a state of injury” (11, p. 21) which in the case of asylum systems refers to actions which appear to help asylum seekers, but instead generate “gradual wounding”, both physical and psychological, including conditions of destitution and endangerment for those seeking sanctuary (10). Forced migrants also struggle to access health and social care in countries of refuge with scholarship indicating that barriers are extensive and include poverty, administrative and communication difficulties (12).

The supplementary distress of palliative care may exacerbate migration trauma by bringing additional uncertainty to those awaiting diagnosis or facing experiences of critical childhood illness and periods of instability whilst living in fear of a child's death (13). Children with palliative care needs have a primary, multifaceted, life-limiting condition where there is no reasonable hope of cure; or life-threatening condition where there may be viable treatments, but these are not always effective (14). Both conditions may result in multi-organ deficiencies and disabilities, requiring many specialists, within hospital in-patient care, respite in hospice or end-of life care and community settings. The term “community setting” refers within this study to care offered within the home or primary care environment, predominately community nursing, general practice services and special school care.

The prevalence of life limiting conditions has increased 2.6 times since 2010, with increased severity of need a consequence of cutting-edge clinical developments, which can prolong the lives of children with the most complex conditions (15). While we know that forced migration has more than doubled in the last decade there is a lack of data regarding the number of forced migrant children requiring palliative care. Poor recording, lack of ethnicity and migrant-status data mean there is no accurate data for the numbers of forced migrant children in receipt of palliative care (15). It is reasonable to assume that these numbers have increased in line with general trends.

Currently children's palliative care (CPC) and forced migration are both neglected in scholarship with gaps in knowledge compounded when combined. A systematic review (16) confirmed the paucity of research in these intersecting areas. Confirming the findings of this review Broom et al. (17) described divergence of beliefs and expectations as a difficult balance between institutional, professional and personal values. The reliance on biomedical priorities by many palliative care professionals (from this point forward, referred to as ‘professionals) were tangible, with conflict surrounding ethics, autonomy and the use of traditional medicines. A failure to recognise or balance competing and conflicting values was seen as potentially damaging to trusting respectful relationships. Conversely, time and everyday interactions were considered important for generating understanding and intimacy between professionals and patients/families. Such encounters included informal exchanges and reciprocal moments of understanding (18).

Guo et al. (19) detail specific aspects of frustration, sadness and desperation post cancer diagnosis for forced migrant families which was compounded by elements of uncertainty. In the Guo et al. study family members actively concealed the truth of diagnosis/prognosis from their relatives to protect them from difficult conversations. Emami and Mazaheri found that the use of family members as interpreters led to information being concealed, which limited professionals ability to share accurate information (20). However, these studies centred around adult care and so specific elements of child-to-parent, parent-to-child and palliative care professional-to-parent/child information giving, receiving and the potential differences between these subsets are absent. Additionally, spirituality and faith were described as life sustaining and enhanced family's ability to cope (21). Whilst faith and community support were optimising to care experienced by patients, these were understandably limited by the separation of forced migration (21).

Palliative Care Professionals caring for children with palliative care needs often describe walking a fine line between professional and personal boundaries, as these become intricately linked through the building of intense long-term relationships (22). However, most challenging for professional's appear to be difficulties in making appropriate end-of-life and symptom management decisions. Abdin et al. (23) detailed the complex and emotional strain of ethical decision making, including the intense support needs of parents. Palliative care professional's detailed moral distress and anxiety when families insisted on continued interventions against the perceived best interests of the child (24). Further, the grief when a child dies resulted in professionals reporting depression (25), guilt (26), sleep disturbances (9), painful memories and reoccurring thoughts (11).

CPC is complex with many ingrained misconceptions. Families' belief systems and grief may lead to a reluctance to engage with specialist services. CPC may also be compromised by a lack of specialist staff, resources and negative perceptions (27, 28). Many parents report clinical levels of distress and fatigue (29), with relentless caregiving and responsibility (30). Long term and community-based trials also detail the effects of physical and social isolation for parents, with a frequent exclusion from the workforce and pervasive grief (31). In addition, there are challenges of controlling physical symptoms like pain and fatigue, communication difficulties with disabled children and active child involvement in decision making and minimisation of suffering (32). A mismatch between the services offered to parents, standards of care and difficulty in gaining or accepting support, are also reported (15).

The complexity and individuality of culture is widely debated (33). Palliative care professional's recognition of migration trauma promotes a holistic focus and identification of ways to address “unique and multidimensional needs” (21). Culture appears integral to individual identity, with the recognition of culture as a non-fixed or discrete notion, considered critical to developing quality, caring relationships (18). Professionals perceptions seem to link to the development of relationships when a forced migrant's lifeworld is understood, but conversely misconceptions can create additional potential for stereotyping.

Forced migrants may struggle to be involved in research projects with fear, suspicion and culturally-related concerns making some individuals reluctant to participate (21). Mistrust of researchers is thought to be significant so overcoming these barriers through ensuring active participation in research seems critical. However, accessibility and inclusivity of the systems and mechanisms for valuing forced migrant voices in research remain inadequate. The limited use of advisory groups, lived experience or patient and public involvement (PPI) within studies involving forced migrants offers a further barrier to their meaningful engagement in research.

When considering the cultural needs for families experiencing palliative care, the report and clinical documentation published by leading CPC Charity Together for Short Lives (18) and The Royal College of Nursing (21) advocates for the importance of communication, religious and spiritual care, with a focus on needs, wishes and beliefs. There has been limited clinical consideration and consequently no guidance produced concerning BME children's palliative care. Looking more widely at guidance around adult palliative care one review within the BME community details problems with access to and provision of palliative care, poor monitoring of ethnicity, limited access to interpretation, mistrust and poor communication with assumptions and stereotypes permeating interactions (34). A report which examined Muslim family's healthcare beliefs with children, families and adult patients also highlighted barriers to accessing care. This included reputation, community expectations, awareness of services, geography, education and confidence (35).

When considering CPC specifically for forced migrant families, poor or limited prognostics, symptom control, resource and accessibility to care in countries of origin, may increase physical and emotional needs. Furthermore, a lack of cultural health capital may mean forced migrant families are not aware of services that exist in their host country and therefore struggle to engage with health care systems and meet professionals' expectations (36). A lack of medical knowledge, vocabulary and communication skills can also contribute to the inequalities observed (37). Presently, there is no data on the numbers of forced migrants with CPC needs and no research into their care or experiences of palliation in the UK. However, there is little doubt that palliative care coupled with forced migration is likely to exacerbate already complex problems and impact upon family health and their care needs. Consequently, this paper addresses the research question “what factors optimise or hinder care for forced migrant children and their families?”.

## Methodology

**Figure 1:**
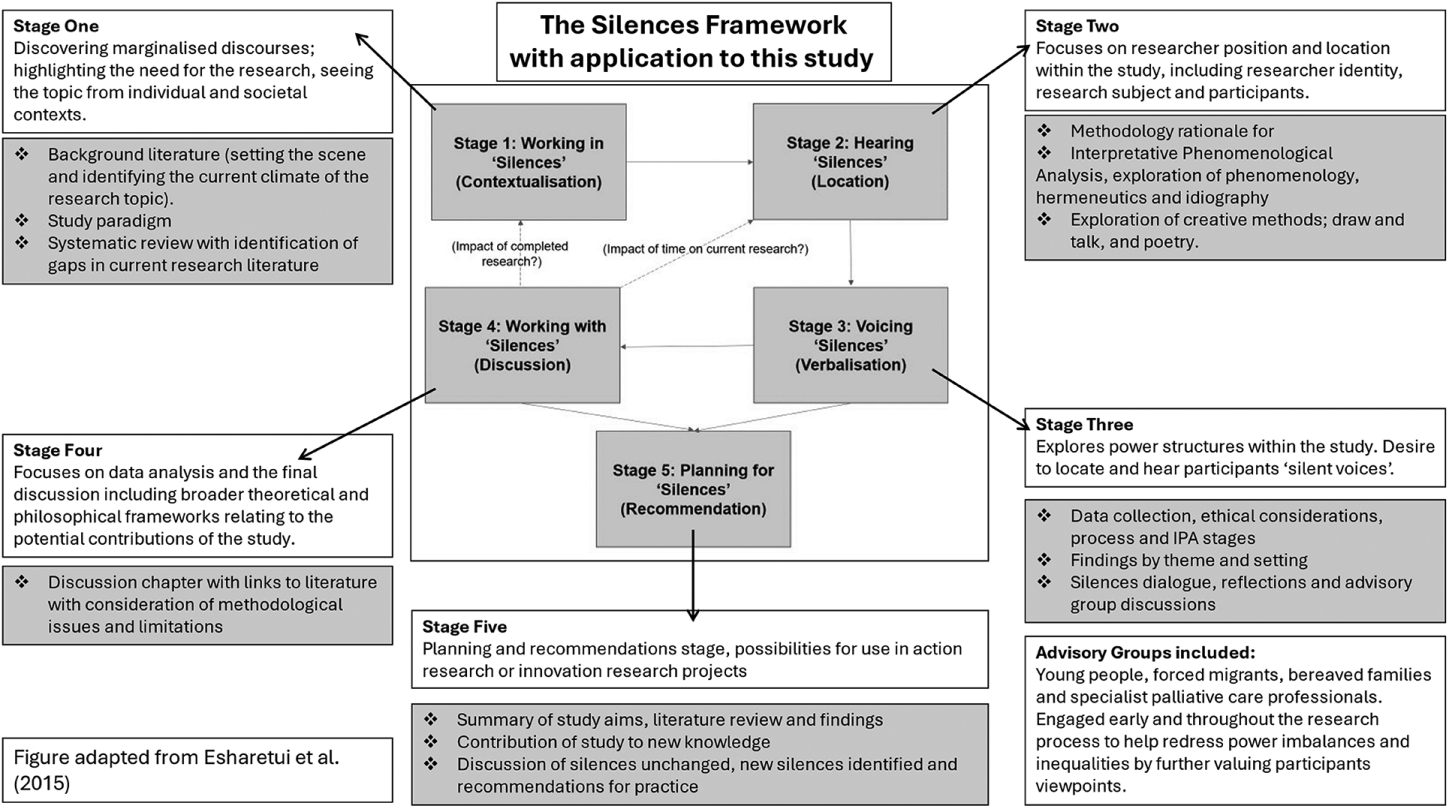
The silences framework and its application within this study.

Qualitative research is concerned with the quality of experience, often asking “what it is like” to experience certain phenomena (38). Interpretative Phenomenological Analysis (IPA) rejects the notion that the world is the same for everyone (39, 40). IPA was used in this study to privilege the voices of participants, by combining the psychological, interpretative and idiographic elements of experience. IPA encourages researchers to view participants with lived experience as experts and thus urges the researcher to prioritise and value individuals. The Silences Framework (41) which underpinned this study, focuses on research issues which are under-researched, silent in policy discourse and poorly prioritised in healthcare practice. The Silences Framework offers an anti-essentialist approach which suggests that research and experience are both ingrained within context, with the researcher playing a central role at all stages of the research study (42). The use of this framework is further clarified within Figure 1.

The Silences Framework was chosen as a philosophical underpinning for this study as it encourages focus on research issues which are under-researched, silent in policy discourse and marginalised from practice, all of which applied to the topic under study. The Silences Framework offered an anti-essentialist approach which suggests that research and experience are both ingrained within context, with the researcher playing a central role at all stages of the research study (39). As such it correlated well with the IPA methodology, which sees interpretation as a fundamental element of how we structure and make sense of our lives.

The Silences Framework revolves around marginalised discourses which are so labelled as they appear undervalued, absent or invisible within policy (43–45). The Silences Framework describes Screaming Silences, which “scream out” to the individuals experiencing them but often need to be uncovered by the outside world and researchers (46). The “listener” is therefore the person who identifies priorities or responds to a particular “silence” (38). Individuals described within such discourse often have little power to act or have a voice in their care. As such they may experience additional inequalities to western families (47). This study aimed to identify the “silences” of forced migrant families in children's palliative care to empower families, create clearer context to aid professionals understanding and debate methods to redress power imbalances. As such six advisory groups contributed throughout this study, to increase insight and influence (45, 48). Advisory group members included children/young people, forced migrants, bereaved parents and specialists in palliative care. Each group participated within their expertise, for example bereaved parents opinions were sought when analysing family participant accounts to increase researcher awareness and a hospital equality group were consulted when considering discrimination and racism within the study. Overall advisory groups collaborated on a pilot study to explore research approaches and questions (palliative care professionals), adaptations on participant information sheets with particular focus on sensitivity, terminology and language use (forced migrant group), joint interviewing (young people) and interview analysis/interpretation (all groups). Last, all advisory group members considered the study findings and advised on the relevance of recommendations.

## Recruitment and sampling

A purposive sampling technique was used to identify and select perspectives and information-rich cases specifically related to the phenomenon of interest (49, 50). Participants were chosen because they offered perspectives of parents/guardians of children requiring palliative care, were children in receipt of care themselves, siblings and professionals having experience of hospital, community and primary care settings. Inclusion and exclusion criteria for the study are included within Table 1.

**Table 1:** Inclusion and exclusion criteria for the study.

Inclusion criteria
- Children aged 2-18 years and their family members who are actively involved in their holistic care - Families six months post bereavement - Children and families who fit the forced migrant definition outlined by UNHCR (2018) and therefore have not come to the UK voluntarily - Children with life-limiting conditions or life-threatening conditions who fall into the categories for palliative care outlined by Together for Short Lives (2018) - Health care workers who are or were actively involved in the provision of care for children and their families and are deemed significant by the families themselves - Children, families, and health care workers within the West Midlands region, who have previously or are actively being care for by the study sites identified - Capacity to consent
Exclusion criteria
- Young adults over 18 years - Perinatal diagnosis of palliative conditions (as these families will require specialist services and have specific needs which warrant separate research) - Economic migrant families (as their needs will defer) - Families whose child may imminently die (as determined by medical professionals) or families who are immediately post bereavement - Children and/or families who do not wish to participate - Children who are deemed too unwell to be approached for interview, their families may still participate if they wish to - Children who are unable to participate for any reason related to their condition, but their families may sill participate if they wish to
These criteria aim for optimal diversity in important characteristics related to the study population and thus offer the inclusion of a wide range of perspectives

To recruit family participants safely, information about the study was first given to palliative care professionals at the two main study sites: a large in-patient children's hospital and a regional children's hospice. These sites were chosen as they work with a large number of children with palliative care needs and have the specialist knowledge and resources available to provide this care. They would also be ideally placed to provide continuing support for families at any point during their involvement in this study. Participant discussion regarding community care was based upon the individual family's utilisation, as this was organised in conjunction with either hospital or hospice care.

## Ethical considerations

The ethical considerations for this study were extensive so we aimed to address both apparent and potentially concealed vulnerabilities and mitigate any risks. There was potential for emotional burden during and following research participation. Potential topics which may have caused distress were handled sensitively and discussed during the pilot interview and with advisory group members. The interviewer was led by the participants' responses to ensure their comfort.

One professional interview and two-family interviews were conducted face to face, with the rest conducted online, at their preference. Pre-interview information was gathered to ensure participant safety (51). Participants were asked if they would be able to talk freely without interruption, in a safe and confidential space and where others could not listen in (children were not present for interviews).

Participants were monitored closely during interviews to recognise distress and to provide appropriate support. Linkages to appropriate services for on-going support were offered to all participants, with the most appropriate sources of support identified by their individual healthcare teams. These included hospital and hospice psychology/counselling services. None of the participants became visibly distressed during their research interviews, but a protocol to manage potential distress was identified.

The consent process was explained verbally prior to the commencement of interviews and reiterated prior to commencement. Participants were made fully aware that whilst their data was to be anonymised and confidential, there were exceptions to this in the event of a safeguarding concern.

## Participants

The recruitment of family members via their usual clinical care teams was undertaken to minimise pressure for families to take part, and ensure they were adequately informed about the project by those with whom they already had a relationship. Care teams were also asked to consider any present physical and mental ill health of the family prior to selection, to help evaluate readiness and ability to participate. An assessment of capacity to consent for all adult participants was also conducted prior to consideration for inclusion in the study. One family were keen to participate but unable due to critical illness.

A pilot interview was conducted with two palliative care professionals experts, prior to participant interviews. This was helpful to discuss questioning strategies and possible prompts for sharing common ground and establishing a trusting relationship (52). Interviews were conducted using an open-ended line of questioning - supported by IPA researchers Smith, Flowers and Larkin (39) who state that it allows the researcher and participant *“to engage in a dialogue whereby initial questions are modified in the light of participants’ responses and the investigator is able to enquire after any other interesting areas which arise”* (p. 57). Examples of interview questions included: Can you tell me a bit about how you came to the UK? And can you tell me about how you first knew your child was unwell?

Interviews commenced at the beginning of 2021, which coincided with the second UK Government stay-at-home/lockdown order to limit the risks of COVID-19. At this time most children had returned to school but there were still limits on social mixing. Fears for children with palliative diagnosis were heightened due to increased medical complexities and increased risk of death should they catch COVID-19 (53). Consequently, many families continued to keep their children at home and were reluctant to visit healthcare environments or have visitors to their homes even after this was permitted by the Government (54). Interviews were thus offered to participants via telephone, online or face-to-face. One family were shielding during recruitment and many professionals’ were limiting their contacts, with some study sites not allowing visitors. Subsequently, only one professional interview and two-family interviews were conducted face to face, with the rest conducted online.

Recruitment was staged to enable families to be identified, their language needs to be assessed and provisions for interpreters sought, and to allow time for adequate reflection and opportunities for questions.

A distinctive feature of IPA is the commitment to a detailed interpretative account of participants lived experiences. Thus, a relatively small sample size is recommended to allow for a rich depth of analysis (55). There are several advantages to undertaking research with small numbers of respondents which include rich insights into specific health experiences (56) and gathering clinical implications (57). Whilst scholars have sometimes criticised the small sample sizes utilised in IPA studies, focusing on a small number of respondents can help to minimise loss of subtle inflections of meaning (58, p. 626), preserve nuances (59) and avoid the researcher becoming overwhelmed by too much data and producing broader, shallow or simply descriptive analysis (60). Larger sample sizes may have created ethical issues, such as overburdening of participants without adequate research gain. When researching families whose children have palliative care needs and the professionals who care for them, multiple layers of insight are required to undertake analysis both individually and collectively. This takes time and is thus not possible on a large scale. The priority in this study was to have data adequacy with the emphasis placed on gathering rich and nuanced accounts which could be learnt from.

## Reflexivity

Reflexive consideration of potential asymmetrical relationships and ways in which they may be exacerbated was important (61). A Cultural Humility stance (62), as advocated for all clinical researchers (63) was thus adopted. At the start of research interactions, the lead researcher (xx) acknowledged her limited knowledge of participants' experiences and established the need for participants to educate her. The five concepts of reflexivity were employed in this study (60). A carefully considered positionality statement gives an introspective account of concern for the research topic and participants. Inter-subjective reflection and discursive deconstruction were used to self-examine the lead researchers influence within research dialogue through a reflexive diary and poetic writing. Mutual collaboration, reflection and social critique were utilised through letter writing, peer correspondence, a Cultural Humility (58) stance in research interviews and through undertaking reflexive dialogue within the research team.

## Analysis

The first step of analysis was to read and listen to each account multiple times whilst making notes of any interesting or relevant details. Next, each participant's account was analysed individually. This single case by case process, involved interpretation of each interview transcript, line-by-line in to get closer to the claims, concerns and understandings of each participant (64). For each transcript, the account of the participant was formatted centrally with large margins on each side for initial exploratory notes on the right-hand side. Exploratory notes included descriptive (descriptions of objects/events/experiences), linguistic (language used, metaphors, repetitions and how words were spoken) and conceptual (interrogative – asking questions of the data) notes (36).

The creation of Experiential Statements (or initial themes) then followed for each participant. This involved an iterative and fluid process of further engagement within each transcript, including processes of reduction, expansion, revision and creativity. This was frequently multi-directional moving from examining part of the transcript text in its own right but also in the context of the whole interview (37). Initially, experiential statements were detailed chronologically and so the links between different portions of the interview could not be clearly seen. To understand the most interesting and important aspects of each participant's account, the experiential statements were printed and cut into sections and then arranged on the floor to break up the ordering. This gave a bird's eye view of the whole account and enabled each experiential statement to be seen as equally important. The experiential themes were then moved around and grouped together, trying different cluster groups until the patterns between statements best represented their interconnections (40). Each cluster of experiential statements was given a title to describe its characteristics, these became the participant's Personal Experiential Themes. Following analysis of all cases, cross-case analysis was conducted to highlight the shared and unique features of experience across participants. The Group Experiential Themes for this study can be seen in Table 2.

**Table 2:** Group experiential themes.

Group experiential themes for family participants	Group experiential themes for HCP participants	Overarching themes
Importance of migration stories and other traumatic experiences	Compassion fatigue, burnout and strategies to overcome	Loss and grief
	Motivation and personal history in relation to role	
	Parental burdens	
Communication and language barriers	Communication and language	Communication
	Relationship building	
	Proactivity, passivity and advocacy	
	Importance of teaching, learning, reflection and education	
Nursing care (optimising and hindering relationships)	Coordination of services and MDT working	
Coping strategies (faith, truth telling and hope, social support and gender roles, parental burdens, love for child, search for normality)	Individualised care	Faith and coping strategies
	Spiritual and religious care	
Poor access to resources	Access and limited health literacy	Alienation and discrimination
Alienation, discrimination and labelling	Racism and discrimination	

## Findings

Fourteen participants were recruited to the study and asked to take part in semi-structured interviews, conducted by xx. Participants included seven family members of three separate families (families Tessa, Ahmed and Khatri). Two families originated from Syria and one from Afghanistan. Seven palliative care professionals including two senior nurses, two social workers, one psychologist, one special school worker and one Muslim Chaplain also took part. All names have been changed to protect participants' identities.

To highlight the main challenges forced migrant families experience when receiving children's palliative care, four overarching themes are explored.

## Loss and grief

For all the families the loss of their homeland coupled with the loss of family and sources of support was detrimental to their wellbeing and coping abilities:

“They used to live as a big family. So even though if mum is not free… can get care from Grandma, from Auntie from Uncle from himself… here there's no family” (Hamza. Father).

For family Tessa the loneliness and isolation they experienced extended to hospital stays in which their teenage son grieved the differences between them and other families:

“He told me nobody come to visit us, we are alone here. Other patients they have visitors, they have friends” (Kinza. Mother).

For all families in this study, the arduous journey to the UK was undertaken to seek a cure for their child/children. Mother Adiva (family Khatri), Father Hamza and Mother Khadija (family Ahmed), described their migration journey and subsequent experiences with the Home Office. When discussing the processing of their asylum claims as derogatory, confusing and frightening:

“He treated me like a homeless person… you know like a beggar… they came and took me in and questioned me… asking loads of dirty questions as in their implying maybe the sex trade… She goes I got really scared then because I thought they were going to sell me (my body) to somebody” (Adiva. Mother).

When considering the impact of migration trauma upon families it was unsurprising that professionals described vicarious trauma and compassion fatigue. Social worker Flo appeared to wrestle with professional boundaries and frequently became protective and concerned for family wellbeing even when off duty:

“I don't mind, you know, calling them after the time that I'm working… I work 9–5 and that's it, but it is, and it isn't because with this family they need that support, then they need to know that they’re supported… but it can be exhausting” (Flo. Social Worker).

Palliative care professionals Flo, Rebecca and Marian, described an often-overwhelming dependency of family members upon individuals. All expressed feeling responsible for families, with Sobia using the metaphor of “carrying her families” through their difficulties and Rebecca using an analogy of feeling like a “one-man band”. Holly gave examples of the longitudinal and traumatic nature of palliative care work and how many lacked the ability or desire to empathise with forced migrant families:

“They’re tired, they just want to do their job, they’re very task orientated and… a white British family who are good fun and laugh with the nurses and easy to get along with, they’re easy to nurse… whereas a family who you don't really understand, possibly because you haven't allowed yourself to understand, the easiest thing rather than to try and learn, is to just distance” (Holly. nurse).

## Communication

All participants discussed the impact that communication and language barriers had upon care. For professionals language barriers affected their ability to understand and be understood by forced migrant families, which was frustrating and restrictive. Families described their inability to communicate with palliative care professionals's leading to diminishing confidence and separation from their care teams. Whilst there was a consensus amongst participants that interpreters were needed and essential, there was also an awareness of financial and dialect restrictions. The awareness that interpreters could not, but should be part of more interactions was acknowledged by professionals and parents:

“They did have interpreters and sometimes there is doctors they speak Arabic so can help but because there are so many hours, you know sometimes the consultant come in different times of the day, interpreter is difficult to bring” (Maaz. Father).

Hamza and Khadija said their experiences with a hospice interpreter were positive, as they were able to have consistent provision and thus build a relationship with her:

“The interpreter, she has made a huge difference, to their lives because, it's not just that she's the interpreter, but she takes a lot of time and I think it's part of her personality to get to know the family” (Flo. Social Worker).

In addition, nurse Holly and Family Tessa described the importance of non-verbal communication. Holly discussed the significance of sitting or kneeling to show intent to stay and listen, the use of silences, touch and eye contact with families. The approach described was of particular importance as many families could not rely on language cues and so had to utilise other elements of non-verbal communication to aid their understanding.

Relationship building and caring concern were also important components. A lack of these elements during her son's hospital admission was described by Mother Adiva. Adiva recounted going without food as nurses assumed she would use the parents' facilities, bring in food or use the canteen. However, all these options were problematic, as she lacked money, confidence in her surroundings and her husband's attention:

“For two months I didn't eat or drink… I said surely there would’ve been a nurse to offer a cup of tea or toast and she said no there was nothing. And she said obviously my husband didn't think let's go home and get her some food” (Adiva. Mother).

Two palliative care professional's described aspects of cultural humility (54) in their relationships with families including mutual respect and two-way learning. A further element conveyed was the importance of kindness and “going the extra mile”. One nurse was described as spending his breaks playing games with Sohail during his chemotherapy, which his family recognised and valued. Both Sohail's siblings said that encountering kindness was essential. The significance of these elements was emphasised when compared to the contrary experiences of hostility, discrimination and trauma forced migrant families encountered.

A final aspect which impacted the ability to provide holistic care, was multidisciplinary and multiagency working. Clara described the difficulties in coordinating community care as she was unable to access patient notes, appointment times or see the care other HCPs had advised in different settings, leading to poor communication between professionals.

## Faith and coping strategies

All three families mentioned their faith (Sikh and Muslim) and how it helped them to cope:

“God will help her… God will always be with you if you be good with your children and look after them” (Khadija. Mother).

Specifically, for Family Tessa, there were incidences in which the professionals differed in their approach to telling Sohail about his palliative diagnosis. The palliative care professionals were said to focus upon swift and direct truth-telling, but lacked understanding of the family's desire to preserve Sohail's faith and hope:

“In the last month of his life the doctor came, and he said sorry Sohail you are dying. You can't imagine this statement to anyone and what about a young boy who is 15, its very, very shock… In our culture, in my country, we never ever tell children this like this” (Maaz. Father).

Professional's misunderstood the family's reluctance as denial. This misunderstanding continued throughout the last week of Sohail's life with an oncology nurse who administered pain relief becoming very graphic in her descriptions surrounding death, using Google Images to aid her, which distressed the family greatly:

“She told him… as a Christian we put bodies in boxes and she Googled and showed him some boxes and graves and a graveyard, it was very, very difficult situation. Sohail very brave and positive and we found him like collapsed” (Maaz. Father).

Family Tessa were further misunderstood when it came to their views on pain. They explained that Sohail's faith helped him to cope with pain and that he saw his pain as a test and that endurance would result in reward:

“We believe that if someone touches the rose and feels the pain this person gets the reward [in the afterlife]… he doesn’t want to take the morphine because he wants to feel the pain and get the reward” (Pasban, sibling).

Similarly, Khadija described an acceptance of her children's condition and rewards for her care accruing, to be received in the afterlife:

“Our God said that whenever I give you, you should be pleased with it and you should like He's gave Bibi as a condition… she's gaining rewards then from God not from people, she's doing it for her faith and for the love for her children” (Khadija. Mother).

Additionally, an all-consuming love for their children was expressed by two mothers in the study as helping them to cope with the fatigue and distress of unyielding caregiving:

“I can't feel where my body ends and hers starts. I feel that her body is a part of mine… her body is a part of my limb and there's no differentiation from where hers stops and mine starts” (Khadija. Mother).

Some professionals were challenged and psychologically tested by forced migrant families who held different beliefs to their own. Different perspectives seemed to confront professionals and highlight the inadequacy of care offered and perhaps contributing poor cultural competence/sensitivity. The resultant feelings of inadequacy appeared to result in professionals becoming defensive, families being problematised and an insistence that families fit with everyday practices.

## Alienation and discrimination

Judgements and assumptions by professionals about forced migrant families appeared common. Family Ahmed discussed a safeguarding referral in which they were accused of deliberate harm to their daughter when she was burnt accidentally:

“The GP speak quickly English like speaking fast so he couldn't understand… when he answered he was worried himself… They tell him to wait outside and (they) do checks on Bibi. I have to wait all day” (Hamza. Father).

Rebecca raised a different issue as she outlined her thoughts regarding entitlement to care. She also discussed the ethics behind parental decisions to bring unwell children to the UK, which she often disagreed with:

“Where it becomes complicated is when there's questions about how families have come into the country, whether they’ve got fraudulent document… we had a high proportion of those families, where actually when you start to piece their journey together, it has lots of question marks” (Rebecca. Nurse).

Rebecca also used some derogatory language to describe family's actions such as: “*strange*”, “*bizarre*” and “*suspicious*”. However, Rebecca seemed insightful into the impact of her limited life experiences, privilege and poor understanding of forced migration:

“I’ve lived a very fortunate life… if I need access to services, I get access to services… so for somebody then to come to me and say they’ve experienced… something horrific… like persecution, I haven't got the skills or the knowledge to understand them” (Rebecca. Nurse).

Further aspects of alienation were described within inconsistencies and inflexibility in the nursing care provided:

“Many times, Sohail asked nurses please let me go to other ward [teenage area] because I have to stay in the bed close to babies crying. So the nurses they say no you are still below 13 he said there is 10 days left and at the same time he surprised when he entered, he found a boy in 5/6 years so you can imagine, how can I answer or reply to my son when he asked me Dad why this 5/6 years, staying (and) me was not allowed to go, there is no answer and no comment” (Maaz. Father).

Additional misunderstandings were evident when professionals appeared to misjudge the expectations placed on forced migrant families, namely, to be proactive and understand the host country's health care system. Many western families have to actively seek responses and follow up from palliative care professionals regarding their children's ongoing care needs (65). Whilst this expectation (rightly or wrongly) may be the norm, Clara appeared to overlook Adiva's limited language skills, low health literary and cultural/gender norms which may have shaped her ability to challenge others:

“It's about being proactive. Maybe giving them (palliative care professionals) a call, but do you (Adiva) know that? Do you know it? And do you? Are you on it? (clicks fingers after each question)… it is frustrating… I think she (Adiva) just thinks this is just her life and she doesn't aspire for more” (Clara. Pastoral Worker, Special School).

Within the examples described, there were many misunderstandings. Families were labelled as unsafe, unworthy, non-engaging, passive or generally problematic. As well as calling these out as discriminatory behaviours, Marian suggested that these assumptions might be an expression of professionals' helplessness and not knowing how to respond appropriately to families' needs. As such further training and awareness of forced migrant family's needs appears imperative.

## Discussion

Within this section a focus on the “silences” (38) uncovered by this study will be given to illuminate the needs of forced migrants in children's palliative care.

The literature surrounding children's palliative care highlights the importance of continuity and consistency in relationships, to help parents cope with the uncertainties of palliative care (66, 67). Within this study families reported that relationship building with palliative care professionals took time but was crucial to the provision of family-centred, holistic care. Families were aware of those professionals who appeared focussed upon “the shift”, compared to those who invested in them. Consistency and reliability of professionals and interpreters were clearly important antecedents to trust and rapport. The use of interpreters during interactions was also seen as optimising. However, existing scholarship on minority children's palliative care describes difficulties in access to interpretation, financial hardship and poor availability of suitable interpreters leading to deferred/rearranged care (68).

Families described the need for professionals to understand their migration experiences to better empathise with them. Scholars have stressed the importance of listening to migration experiences to better understand individuals past traumas and family context (69). However, such conversations may prove difficult and can be challenging for professionals to pitch correctly, which can lead to avoidance. A “silence” revealed through this study was families clear desire to share their individual experiences. Social worker Flo noted that Family Ahmed had revealed more about their previous experiences during their interview than in her two-year relationship with them. As well as the importance of sensitive communication, another consideration concerns the timing of such conversations. Professional experiences and those of migration scholars lead us to suggest that the sharing of migration journeys would ideally occur in the first few meetings to gather context and show caring concern.

The migration trauma and ongoing difficulties experienced by forced migrant families may also explain the importance of feeling cared for and the impact when palliative care professionals went the extra mile to support them (70). Often the elements described were subtle, such as smiling and greeting families, spending extra time with them and being accessible. Families were aware when they were being shown caring concern and when this was lacking. Professionals' body language, tone of voice and their authenticity within interactions appeared crucial (71). Good information flow is vital, including regular updates and “checking in” with families to build relationships and meet evolving needs (59). This concurs with research about refugees with advanced cancer, their caregivers, and health care professionals in Jordan (72). For forced migrant families in this study, these regular check-ins were often missed or inadequate. The expectations, personal values and goals of care were thus often undiscussed or misunderstood. A further “silence” uncovered during this study was that poor experiences between families and palliative care professionals often caused parents to feel abandoned and to question the accuracy of professional's information. Davies et al. (61) state that suboptimal patterns of information sharing can contribute to frustration, anger and sadness.

Further elements of misunderstanding and minimising of family's beliefs were seen surrounding truth telling and hope. A workshop of experts in CPC discussed that challenges arose when there was conflict or if decision-making processes felt “foreign” (73). However, professionals may need to sit with conflict and “*be more comfortable with messy*” to facilitate culturally sensitive care (63, p. 849). It was also suggested that when differences feel hard to navigate for professionals, there are often implicit or explicit biases and corresponding assumptions present (63).

A further contribution of this study surrounds the uncovering of a “silence” within the provision and understanding of spiritual care to families and the impact of terminology. Family Tessa's Muslim faith was of paramount importance to them. However, their spiritual needs were often not valued or even dismissed by professionals. Their nurse, Holly, described nurses interrupting prayer and a general lack of understanding around the family's desire to allow their son Sohail to live out his faith, particularly towards the end of his life. This may link to misunderstandings and poor explanations of palliative care terminology. In much of the literature, myths surrounding barriers to palliative care are detailed. One such “myth” is a perception by ethnically diverse families that “suffering makes you stronger” (74, p. 168). By using the word myth rather than belief, the language indicates that beliefs about pain are a falsity which needs to be dispelled, rather than a religious belief to be respected and understood. Challenges around understanding religious belief and pain are thus evident, as the link between suffering, strength and eternal favour is advocated in many religions including Christianity, Islam, Buddhism and Hinduism.

The link between cultural practices and health beliefs was not always understood by professionals (66). The lack of education around faith and spirituality is emphasised by Fowler (75) who states that for those with a strong faith, religious obedience is intentional and “*faith is a matter of life and breath*” (p. 391). Scholars describe limited professional education on spiritual care and a consequential lack of skill and confidence (76, 77). Timmins (70) highlights challenges with assessing the spiritual needs of families with prerequisite for self-awareness and openness to different levels of dialogue.

Many of the aspects discussed by family participants appear to link to a simplistic understanding of their culture by palliative care professionals and a lack of ability to provide individualised care. The idea that culture is static and can be learnt and repeated for groups, may derive from traditional training methods such as Cultural Competence (78). Cultural competence has been criticised for giving professionals an overestimation of personal cultural competence levels which may encourage arrogance or led to ethnic reductionism (79) and precipitate cultural misunderstandings. Families talk about culturally sensitive care, connected closely to the philosophy of Cultural Humility (61) in which mutual respect was observed, listening and time was given to families and professionals were seen as open and able to acknowledge their limitations, whilst learning from and with families. These findings offer insights into how cultural sensitivity may be improved for forced migrant families when the Cultural Humility (61) rather than Cultural Competence model (78) is followed.

The lack of social support and its negative impact upon families described in this study was illuminating but unsurprising, particularly with the additional complexity of the COVID-19 pandemic. The pandemic meant many palliative care professionals were redeployed and the support available was reduced. Whilst this was challenging for all families, the implications may have been more severe for forced migrant families who did not have an outside support network. The absence of social support for forced migrant families may differ to that available for established black ethnic minority families in palliative care, who may have additional family, faith and/or community support. This difference may have created confusion for professionals and an additional “silencing” or lack of understanding of the desperate need for support described by forced migrant families.

The burdens that families endured when caring for their children were eased by normality, such as school life and the utilisation of non-clinical spaces for family time-out where possible. Parents often experienced hypervigilance, with a feeling of being on duty 24 h a day (80) and so rest and appropriate respite (including hospice stays) were beneficial. However, the responsibility, duty and intense love parents had for their children needed to be balanced with trusting palliative care professionals to care for their children as they would at home. For forced migrant families, this may be compounded by the precious nature of their children which saw all three families uproot their lives at great personal cost, to better meet their children's needs.

Within this study, elements of burnout were discussed by professionals. The long-term effects of witnessing intense suffering on palliative care professionals is recognised (81) but within this study there were also elements of disillusionment and emotional blunting. Markey (82) discusses disengagement strategies used by professionals when caring for ethnically diverse patients and suggests that they may take painstaking measures to rationalise insensitive care. An inability to challenge behaviour and poor care, places palliative care professionals at risk of becoming detached and ineffective (83). Conversely, the development of moral reasoning may act as a process for discerning ethical action (73). Palliative care professionals may thus become empowered and able to challenge associated social injustices and health inequalities (84).

A lack of access to services, hospice care, inappropriate housing, inadequate financial support and a lack of healthcare literacy appeared to hinder care. Problems with access to services and funding are commonplace in the literature surrounding refugee health (85). For families in this study, much of this complexity came from poor understanding of entitlement and eligibility. Further challenges came from the medical complexity of palliative care with the need for multiple professionals and organisations/settings for the provision of care. Subsequently, multidisciplinary and multiagency working can be challenging for staff and families to navigate (86). For forced migrant families, this is compounded by communication difficulties and a lack of understanding of roles/responsibilities. However, co-ordinated multidisciplinary team working can enhance support, increase expertise and provide invaluable insights and peer support during challenging cases (87).

## Limitations

Due to the complexity of disability and the limitations of the pandemic, none of the cared-for children at the centre of this study were able to share their experiences of care. Thus further research specifically focusing on children's perspectives is recommended.

Whilst online interviewing was used with many participants in this study, the limitation of missing non-verbal cues was minimised with the use of video functionality and additional researcher attentiveness (88). The timing of the research interviews meant many of the participants had already become familiar and comfortable with online software, which beneficial and minimised user errors often cited as disadvantages to online interviewing (76). Further, Hanna and Mwale (89), suggest benefits to online mediums, in that they may offer a more comfortable environment to share personal experiences, can be more convenient and give more control to participants, whilst also offering easy data capture with built-in recording programmes.

Researcher bias in an interpretative study is somewhat inevitable, however this was reduced by continual input from advisory group members throughout the study and by using many methods of reflexivity to aid self-awareness and prompt transparency of methods.

The Silences Framework has limitations; the relatively new nature of the framework makes critique of its application difficult. However, one study reflects on the benefit of the Silences Dialogue and Collective Voice's (PPI) processes (90). These cyclical data analysis phases can be used to stimulate member checking by those independent to the study but with important lived expertise (91). In this study, the Silences Dialogue stage was helpful in valuing and implementing advisory group feedback. However, this stage can lead to confusion (78), thus careful explanation is needed.

## Recommendations

The findings of this study suggest limited palliative care professionals knowledge and understanding of forced migration experiences and trauma. The development of a specific professional education pathway on forced migration is thus recommended. Topics may include the inclusion of vignetters based on lived experiences to increase knowledge/empathy and self-awareness/bias education alongside moral reasoning training. Additionally, the inclusion of spiritual care education is crucial to meeting need and gaining further knowledge of belief systems and their importance to families.

Educational techniques to instil such practices may include development of pedagogical approaches such as stories, narratives and participant poetry, film and infographics, scenarios, role play and drama. These aspects may aid critical thinking, offer multiple perspectives for debate, and support the identification and assessment of complex needs. In conjunction with such pedagogy the inclusion of interdisciplinary training with the involvement of faith leaders and refugee specific organisations to share expertise and build supportive relationships for future practice is also recommended.

Clinical recommendations include provision of additional time and consideration of staff allocations, to build relationships and offer continuity to families. Additional time available to support forced migrant families may aid professionals in navigating complexity such as addressing legal and financial needs, whilst also sensitively managing interactions with interpreters. It is also advised that addressing communication difficulties be prioritised by health and social care organisations, with additional funding for interpreters, their training and further understanding of the interpreter role and ability to provide family support.

The importance of continued and sustained collaboration with advisory group expertise during research studies was highlighted by this study. Advisory group input was extremely valuable in helping the researchers to shape the study, understand participant perspectives and to develop appropriate recommendations. Ziersch et al. (92) concur stating that a central aspect of research with forced migrant communities is that of close community collaboration to ensure validity.

A further practice recommendation is the prioritising of respite for forced migrant families with the aim of providing additional support, normality and limiting the isolation often experienced. Lastly the provision of palliative care professional wellbeing support is crucial to addressing the impact of burnout and compassion fatigue described in this study.

## Conclusion

In conclusion, the literature appraised for this study illuminated many “silences” in both the fields of children's palliative care and forced migration. This study has brought the two fields together to reveal unique insights.

The findings of this study indicated limited palliative care professional knowledge and understanding of forced migration experiences, spirituality and trauma so the development of an education pathway is recommended. Topics discussed would serve to emphasize the role of lifelong learning in producing humble practitioners who want to redress power imbalances across professions and within their relationships with families.

Additional clinical time, interpretation provision and professional support to prevent burnout and compassion fatigue is essential. Continued research in this area is crucial to understanding forced migrant families' perspectives. Research which addresses the challenges of understanding the views of disabled and non-verbal children is important to ensure their views are captured. Finally, and perhaps most importantly is the need for care, which is compassionate and humane, and takes account of individual forced migrant family's needs and experiences. Father Maaz articulates:

“They have to know they are dealing with a human, not a machine. They are not repairing a machine, this is a human, he has feelings, with heart”

Each palliative care interaction will create lasting memories for families particularly those whose children are at end-of-life. Palliative care professionals have a duty to provide equitable care and thus need to consider the additional needs of forced migrant families to ensure their holistic needs are met and their experiences are positive.

## Data Availability

The datasets presented in this article are not readily available because rare conditions may make patients identifiable. Requests to access the datasets should be directed to m.w.clancy@exeter.ac.uk.
